# Safety of in-hospital delay of appendectomy in complicated appendicitis – a propensity score–matched analysis of 1027 patients

**DOI:** 10.1007/s00423-026-04264-3

**Published:** 2026-10-23

**Authors:** Lena Anthuber, Michael Hoffmann, Stefan Schiele, Gernot Müller, Andrea Mair, Florian Sommer, David Pinto, Matthias Anthuber, Katharina Beyer, Matthias Schrempf

**Affiliations:** 1 Department of General, Visceral and Transplantation Surgery, University Hospital Augsburg, Stenglinstrasse 2, Augsburg, 86156 Germany; 2 Department of Computational Statistics and Data Analysis, Institute of Mathematics, University of Augsburg, Augsburg, Germany

**Keywords:** Complicated appendicitis, Surgical timing/in-hospital delay, Appendiceal perforation, Postoperative complications, Risk factors, Propensity-score matching

## Abstract

**Introduction:**

Appendectomy is one of the most frequently performed emergency surgical procedures worldwide. Current guidelines recommend surgical intervention within 24 h for uncomplicated appendicitis; however, the optimal timing for patients with complicated appendicitis remains unclear. Recent studies suggest that in-hospital delay may be safe in uncomplicated cases, yet patients with clinical or radiological signs of perforation are usually excluded. This study aims to identify clinical and laboratory risk factors for perforation and to evaluate whether it is safe to delay surgery by 6 to 9 h in patients presenting with signs of complicated appendicitis.

**Methods:**

This retrospective single-center study included patients who underwent emergency surgery for complicated appendicitis between January 2008 and June 2023. Perforation was defined histopathologically, and complications were recorded perioperatively. Multivariable logistic regression was used to identify independent risk factors for perforation and complications. Propensity-score (PS) matching was performed to control confounding factors.

**Results:**

A total of 1,027 patients with suspected complicated appendicitis were included. PS matching yielded two cohorts of 327 patients. No significant differences were observed in perforation rates (46.5% vs. 47.1%; *p* = 0.88) or complication rates (23.5% vs. 21.4%; *p* = 0.51). Logistic regression analysis of the PS matched groups confirmed that a delay of surgery of > 6 h was not associated with increased risk of perforation (OR 1.03; 95% CI 0.75–1.41; *p* = 0.87) or complication rate (OR 0.87; 95% CI 0.59–1.27; *p* = 0.47). A 9-hour threshold to surgery showed no significant differences in perforation rates (50.0% vs. 44.9%; *p* = 0.34) or complication rates (24.7% vs. 19.7%; *p* = 0.25). Logistic regression analysis did not demonstrate an association between a surgical delay of > 9 h and either perforation (OR 0.79; 95% CI: 0.51–1.22; *p* = 0.28) or complication rate (OR 0.68; 95% CI: 0.39–1.17; *p* = 0.16).

Age > 65 years, higher BMI, symptom duration > 48 h at the time of admission, elevated inflammatory markers, and suspected perforation on imaging were identified as independent risk factors for perforation. Higher ASA scores, male sex, suspected perforation on imaging, and elevated CRP levels were associated with increased complication rates.

**Interpretation:**

A delay in surgical intervention of 6–9 h may be acceptable in patients with suspected complicated appendicitis without increasing the risk of perforation and / or complications.

**Supplementary Information:**

The online version contains supplementary material available at https://doi.org/10.1007/s00423-026-04264-3.

## Introduction

Appendectomy is one of the most frequently performed emergency surgeries worldwide [1]. Although the use of antibiotics in cases of uncomplicated appendicitis is debated, surgery is the gold standard for complicated appendicitis [2, 3]. Although most cases of appendicitis are uncomplicated, a significant proportion of patients present with perforated appendicitis [1]. Perforated appendicitis is associated with longer hospital stays, higher complication rates, and increased mortality compared to uncomplicated appendicitis. Signs of perforation can be detected through imaging or elevated inflammatory markers, such as white blood cell count and/or C-reactive protein levels [1, 4].

The recently updated 2025 World Society of Emergency Surgery (WSES) Jerusalem Guidelines confirm that appendectomy for uncomplicated appendicitis may be safely delayed within 24 h without increased risk of adverse outcomes, and continue to endorse laparoscopic appendectomy as the standard surgical approach [3]. Furthermore, the 10-year follow-up of the APPAC randomized clinical trial reported a cumulative appendectomy rate of 44.3% among patients initially managed with antibiotics alone for uncomplicated appendicitis, reinforcing that surgery remains the definitive treatment and that the question of operative timing is highly clinically relevant [5]. Retrospective analyses indicate that patients with longer in-hospital delays tend to have higher rates of perforation and complications such as surgical site infections [2, 6–9]. However, large retrospective studies and meta-analyses as well as the prospective, multicenter PERFECT and DELAY trials suggest that a waiting time of 24 h does not lead to a higher perforation rate in patients without preoperative signs of complicated appendicitis [6, 10–12]. These findings may be biased, as patients with a more acute clinical presentation, significant laboratory abnormalities, or imaging evidence of perforation are more likely to undergo surgery earlier. Despite advances in diagnostic methods, predicting which patients are at higher risk for perforation continues to be a challenge. The specific risk factors for perforation and complications have yet to be fully identified.

In clinical practice, high workload and limited capacities often lead to a delay in surgical treatment of emergent cases. In smaller hospitals, a qualified surgeon may not always be available, while in larger, high-volume centers, more urgent cases may take priority. Furthermore, the potential risk of nighttime operations needs to be considered as surgeon fatigue is associated with higher error rates [13]. Although most patients undergo surgery within 24 h, the question remains whether this timeframe is safe for those showing signs of perforated appendicitis. In the literature, this cohort is often excluded from analysis. Obtaining prospective data may be challenging, as it is ethically difficult to justify a delay in surgery for patients presenting with signs of complicated appendicitis. Therefore, we performed a propensity-matched analysis of retrospective data to adjust for clinical, laboratory and radiologic signs that would usually prompt an early surgical intervention to reduce selection bias.

The goal of this study was to investigate whether an in-hospital delay of surgery is associated with a higher risk for complications and perforation in patients presenting with signs of perforated appendicitis.

## Methods

This retrospective single center study was conducted at the Department of General, Visceral and Transplant Surgery at the University Hospital Augsburg.

The study protocol was approved by the ethics committee of the Ludwig-Maximilians-University (LMU), Munich (reference number 23–0500) and the study was conducted in accordance with the Declaration of Helsinki.

### Study population and definitions

Patients who underwent emergency surgery for suspected appendicitis between January 2008 and June 2023, who were aged 16 years or older at the time of presentation and had either a significantly elevated C-reactive protein level (≥ 100 mg/l) and/or suspected perforation on preoperative imaging were included for analysis. The CRP threshold of ≥ 100 mg/l was selected based on published evidence supporting its high specificity for complicated appendicitis [14, 15]. The CRP criterion was used solely as an inclusion criterion to identify patients at elevated preoperative risk.

Data were retrospectively obtained from the hospital’s clinical information system. Perioperatively documented variables such as age, body mass index (BMI), symptoms at the time of presentation, duration of symptoms, laboratory and radiographic findings, American Society of Anesthesiologists (ASA) score, surgical data, perioperative complications, and length of hospital stay were reviewed and entered into an electronic database for statistical analysis.

Duration of symptoms was documented at the time of initial clinical assessment by the treating physician and recorded in the hospital’s electronic clinical information system as part of the routine emergency surgical admission history. The time categories used for surgical delay (≤ 6 h, > 6 h, and > 9 h from diagnosis to surgery) were chosen to reflect clinically relevant triage intervals: the 6-hour threshold aligns with benchmarks established in prior meta-analyses and retrospective studies on in-hospital delay.

If the radiologist or physician had documented a perforation or suspected perforation in the written imaging report, a “suspected perforation on imaging” was documented. If the histopathologic examination revealed a perforation or gangrene in the appendiceal specimen, the outcome “perforation” was documented. The complication rate was defined as the percentage of patients in whom a perioperative complication occurred. Complications were classified according to the Clavien–Dindo classification system and the Comprehensive Complication Index. Patients treated with antibiotics and/or interventional drainage with surgery were not included in this study.

### Surgical intervention

Open appendectomy was performed through an oblique McBurney incision using a muscle-splitting technique. Laparoscopic appendectomy was performed with three trocars (one 10-mm trocar for the camera, one 5-mm trocar, one 12-mm trocar). If conversion from laparoscopy to open surgery was necessary, either a midline incision or an oblique incision in the right lower quadrant was performed, depending on the intraoperative findings and at the discretion of the surgeon in charge. For laparoscopic procedures, the base of the appendix was transected using a laparoscopic stapler. In open procedures, either a stapler was used to dissect the appendix, or the base was ligated with subsequent dissection and inversion of the appendix stump.

### Statistical analysis

Continuous data is presented as mean ± standard deviation (SD) or median with interquartile range, depending on distribution. Categorical data is presented as numbers with percentages. Continuous variables were compared using the independent t-test and the Mann-Whitney-U test depending on distribution. Categorical data was compared using the χ2 test. A two-sided *P* < 0.05 was considered significant. Logistic regression was used to adjust for *American Society of Anesthesiologists* (ASA) score, age, diabetes, intake of anticoagulants, previous abdominal surgery, body mass index (BMI), white blood cell (WBC) count, CRP level, presence of guarding on physical examination, duration of symptoms and suspected perforation on imaging. Odds ratios with a 95% confidence interval (CI) were reported for each risk factor.

A matched propensity score (PS) analysis was performed to account for differences between patients with a shorter and a longer time from diagnosis to surgery. The PS was estimated using a logistic regression model with variables recorded during admission: age, WBC count, CRP level, duration of symptoms, gender, presence of abdominal guarding on physical examination, ASA score, BMI, suspected perforation on imaging, diabetes, previous abdominal surgery and intake of anticoagulants.

Patients with a longer time from diagnosis to surgery and those with a shorter time were matched 1:1 on the logit-transformed PS by a greedy nearest neighbor algorithm with a caliper of 0.2 of the SD of the logit of the PS. Balance between both groups was judged by standardized differences, where a satisfying matching is obtained when the standardized mean difference (SMD) is less than 0.1 for all variables. We reported the percentage of perforation and complication in each group after the matching and used a χ2 test to compare those. Further, we performed a logistic regression in the matched sample to adjust for remaining differences between the matched groups and calculated odds ratios with a 95% confidence interval for each endpoint.

Statistical analyses were undertaken using SPSS^®^ for macOS^®^, version 29 (IBM, Armonk, New York, USA) and R, version 4.3.1 (R Foundation for Statistical Computing, Vienna, Austria).

## Results

Between January 2008 and June 2023, a total of 1,027 patients presented with suspected appendicitis and either markedly elevated CRP levels (≥ 100 mg/l) and / or perforation on diagnostic imaging, suggesting a complicated or more advanced appendicitis. In this patient population the mean age was 49.3 years, and 251 (24.4%) patients were older than 65 years. The majority (77%) of the study population presented with symptoms lasting for more than 24 h. The mean waiting time until surgery was 6.8 h (SD ±7.2) and 87.9% of the study population underwent surgery within 12 h. Mean leukocyte count was 14.6/nl (SD ± 4.8) and mean CRP level was 158 mg/l (SD ± 83). Perforation was suspected preoperatively in 41.0% (*n* = 414). Laparoscopic surgery was completed in 813 (79.2%) patients. In 148 cases (14.4%) laparoscopic surgery was attempted but conversion to open surgery was required. Primary open appendectomy was performed in 66 (6.4%) patients. Histopathological examination revealed gangrenous or perforated appendicitis in 47.5% (*n* = 488) of all cases. A perioperative complication occurred in 250 (24.3%) patients and 4 (0.4%) patients died during the postoperative course. Median postoperative length of hospital stay was 4.3 days. The demographic characteristics are shown in Table 1.

**Table 1:** Baseline demographic characteristics

Characteristic	*n* = 1,027
Type of surgery Laparoscopic Open Conversion from laparoscopic to open	813 (79.2%) 66 (6.4%) 148 (14.4%)
ASA Score 1 2 3 4 / 5	423 (41.2%) 425 (41.4%) 169 (16.5%) 10 (1.0%)
Age (mean, SD)	49.3 (±19.6)
Sex Female Male	497 (48.4%) 530 (51.6%)
BMI (mean, SD)	26.8 (±5.6)
Diabetes	48 (4.7%)
Use of anticoagulants or platelet inhibitors	249 (14.5%)
Previous abdominal surgery	158 (15.4%)
Abdominal guarding	525 (51.1%)
Duration of symptoms > 48 h	512 (52.9%)
Preoperative leukocyte count (per nl) Mean (SD)	14.6 (4.8)
Preoperative CRP level (mg/l) mean (SD)	158 (83)
Preoperative imaging Ultrasound CT MRI	984 (95.8%) 292 (28.4%) 1 (0.1%)
Suspected perforation on imaging	414 (41.0%)
Diagnosis to surgery (hours) mean (SD) ≤ 6 h > 6 h > 9 h	6.84 (±7.2) 673 (65.5%) 354 (34.5%) 186 (18.1%)
Operating time, minutes (median, (IQR))	70 (55–92)
Perforation on histopathologic examination	488 (47.5%)
Complication rate	250 (24.3%)
Complication Clavien-Dindo ≥ 3	71 (6.9%)
Comprehensive Complication Index (CCI)^®^	5.7 (±12.3)
Negative appendectomy	85 (8.3%)
Mortality	4 (0.4%)
Length of postoperative stay, days (median, (IQR))	4.3 (2.7–6.5)
Date are mean ± SD or n (%) or median (IQR) BMI body mass index, ASA American Society of Anesthesiologists, CRP C-reactive protein	

### Logistic regression analysis

Multivariable logistic regression analysis adjusting for potential risk factors was performed for the endpoints “perforation” and “perioperative complication”. Risk factors for a perforation were age > 65 years (OR 1.50; 95% CI 1.04–2.17; *p* = 0.029), a higher BMI with an OR of 1.14 per every 5 points in increase of BMI (95% CI 1.002–1.29; *p* = 0.049), a duration of symptoms > 48 h (OR 1.45; 95% CI 1.11–1.91; *p* = 0.007 at the time of admission, suspected perforation on preoperative imaging (OR 1.92; 95% CI 1.46–2.54; *p* < 0.001), a leukocyte count > 15/nl (OR 1.63; 95% CI 1.24–2.16;*p* < 0.001) and CRP levels > 150 mg/l (OR 1.60; 95% CI 1.21–2.12; *p* < 0.001).

Higher ASA scores (OR 1.51; 95% CI 1.17–1.94; *p* = 0.001), male sex (OR 1.57; 95% CI 1.13–2.18; *p* = 0.007), suspected perforation on preoperative imaging (OR 1.55; 95% CI 1.12–2.14; *p* = 0.008) and CRP levels > 150 mg/l (OR 1.89; 95% CI 1.36–2.64; *p* < 0.001) were associated with increased risk for complications in the multivariable analysis.

An in-hospital delay > 6 h in was neither associated with an increased risk for perforation (OR 0.99; 95% CI 0.75–1.32; *p* = 0.97) nor an increased risk for complications (OR 0.81; 95% 0.58–1.13; *p* = 0.22). The results of the multivariable analysis are shown in Tables 2 and 3.

**Table 2:** Risk factors for perforation in patients with CRP ≥ 100 mg/l and / or suspected perforation – multivariable analysis

Risk factor	OR	95% CI	*p* (multivariable)
ASA-Score	1.08	0.86–1.35	0.503
Age > 65 years	1.50	1.04–2.17	**0.029**
Male sex	0.97	0.74–1.28	0.85
BMI*	1.14	1.002–1.29	**0.049**
Diabetes	0.88	0.45–1.70	0.69
Previous abdominal surgery	1.23	0.84–1.81	0.29
Symptom onset > 48 h	1.45	1.11–1.91	**0.007**
Abdominal guarding	0.96	0.74–1.26	0.79
Suspected perforation on imaging	1.92	1.46–2.54	**< 0.001**
Leukocyte count ( /nl) ≤ 15 > 15	- 1.63	- 1.24–2.16	- **< 0.001**
CRP level (mg/l) ≤ 150 > 150	- 1.60	- 1.21–2.12	- **< 0.001**
In-hospital delay > 6 h	0.99	0.75–1.32	0.97

*BMI* body mass index, *ASA* American Society of Anaesthesiologists, *CRP* C-reactive protein

* per 5 units increase in BMI

**Table 3:** Risk factors for a perioperative complication in patients with CRP ≥ 100 mg/l and / or suspected perforation – multivariable analysis

Risk factor	OR	95% CI	*p* (multivariable)
ASA-Score	1.51	1.17–1.94	**0.001**
Age > 65 years	1.43	0.96–2.12	0.078
Male sex	1.57	1.13–2.18	**0.007**
BMI*	0.94	0.81–1.09	0.44
Diabetes	1.27	0.63–2.51	0.49
Previous abdominal surgery	1.43	0.93– 2.19	0.10
Symptom onset > 48 h	1.21	0.88–1.67	0.25
Abdominal guarding	0.88	0.64–1.20	0.41
Suspected perforation on imaging	1.55	1.12–2.14	**0.008**
Leukocyte count ( /nl) ≤ 15 > 15	- 1.21	- 0.88–1.68	- 0.24
CRP level (mg/l) ≤ 150 > 150	- 1.89	- 1.36–2.64	- **< 0.001**
In-hospital delay > 6 h	0.81	0.58–1.13	0.22

*BMI* body mass index, *ASA* American Society of Anaesthesiologists, *CRP* C-reactive protein

* per 5 units increase in BMI

### Propensity score matched analysis

Propensity score matching was performed to compare patients who waited 6 h or less until surgery with patients who waited more than 6 h. The matching resulted in two groups of 327 patients each. The standard mean difference (SMD) for baseline characteristics before and after matching indicates successful matching of the two groups. The baseline characteristics and SMD values before and after matching are shown in Tables 4 and 5. In the matched data set, perforation rates did not differ between patients with an in-hospital delay of 6 h or less and more than 6 h (46.5% vs. 47.1%; *p* = 0.88). The complication rate was 23.5% in patients with a waiting time ≤6 h and 21.4% in patients with a waiting time >6 h (*p* = 0.51). A doubly robust logistic regression analysis which included the variables used for matching was performed. In the logistic regression analysis in the matched data set, a waiting time of more than 6 h was not associated with an increased perforation rate (OR 1.03; 95% CI 0.75–1.41; *p* = 0.87) or complication rate (OR 0.87; 95% CI 0.59–1.27; *p* = 0.47).

**Table 4:** Baseline characteristics before matching in patients with CRP≥100 mg/l and / or suspected perforation

	Time to surgery ≤ 6 h	Time to surgery > 6 h	*p*	SMD
	*n* = 620	*n* = 332		
Age	48.5 (19.4)	49.3 (19.7)	0.567	0.039
Leukocyte count > 15/nl	279 (45.0%)	121 (36.4%)	0.013	0.175
CRP level > 150 mg/l	279 (45.0%)	162 (48.8%)	0.293	0.076
Duration of symptoms			0.136	0.182
0–12 h	39 (6.3%)	25 (7.5%)		
12–24 h	106 (17.1%)	51 (15.4%)		
24–48 h	161 (26.0%)	65 (19.6%)		
48–72 h	111 (17.9%)	62 (18.7%)		
more than 72 h	203 (32.7%)	129 (38.9%)		
Sex male female	330 (53.2%) 290 (46.8%)	159 (47.9%) 173 (52.1%)	0.133	0.107
Abdominal guarding	363 (58.5%)	144 (43.4%)	< 0.001	0.307
ASA score	1.75 (0.74)	1.77 (0.75)	0.724	0.024
BMI	27.0 (5.7)	26.5 (5.7)	0.262	0.076
Suspected perforation on imaging	268 (43.2%)	125 (37.7%)	0.110	0.114
Diabetes	24 (3.9%)	20 (6.0%)	0.178	0.099
Previous abdominal surgery	82 (13.2%)	65 (19.6%)	0.013	0.172

Date are mean ± SD or n (%)

*SMD* standard mean difference, *BMI* body mass index, *ASA* American Society of Anaesthesiologists, *CRP* C-reactive protein

**Table 5:** Baseline characteristics after matching in patients with CRP≥100 mg/l and / or suspected perforation

	Time to surgery ≤ 6 h	Time to surgery >6 h	*p*	SMD
	*n* = 327	*n* = 327		
Age	49.3 (19.7)	49.1 (19.7)	0.907	0.009
Leukocyte count > 15/nl	124 (37.9%)	120 (36.6)	0.808	0.025
CRP level > 150 mg/l	155 (47.4%)	159 (48.6%)	0.814	0.024
Duration of symptoms			0.918	0.076
0–12 h	24 (7.3%)	24 (7.3%)		
12–24 h	53 (16.2%)	51 (15.6%)		
24–48 h	66 (20.2%)	64 (19.6%)		
48–72 h	69 (21.1%)	62 (19.0%)		
more than 72 h	115 (35.2%)	126 (38.5%)		
Sex male female	159 (48.6%) 168 (51.4%)	156 (47.7%) 171 (52.3%)	0.876	0.018
Abdominal guarding	139 (42.5%)	144 (44.0%)	0.752	0.031
ASA score	1.75 (0.75)	1.76 (0.74)	0.875	0.012
BMI	26.8 (5.7)	26.5 (5.7)	0.560	0.046
Suspected perforation on imaging	120 (36.7%)	124 (37.9%)	0.808	0.025
Diabetes	16 (4.9%)	18 (5.5%)	0.860	0.028
Previous abdominal surgery	59 (18.0%)	60 (18.3%)	1.0	0.008

Date are mean ± SD or n (%)

*SMD* standard mean difference, *BMI* body mass index, *ASA* American Society of Anaesthesiologists, *CRP* C-reactive protein

To assess if an even longer delay until surgery in patients with markedly elevated CRP levels or suspected perforation poses a risk for perforation or complication propensity score matching was repeated to compare patients with an in-hospital delay of 9 h or less and more than 9 h. The matching resulted in two groups consisting of 178 patients each. The groups showed a high level of equality with respect to variables used for the matching as indicated by SMD values for the baseline characteristics before and after the matching (Supplemental Table 1a and 1b). Perforation rates (50.0% vs. 44.9%; *p* = 0.34) and complication rates (24.7% vs. 19.7%; *p* = 0.25) did not differ between patients who waited ≤ 9 h and >9 h in the matched study population. Logistic regression analysis in the matched study population did not reveal an association between an in-hospital > 9 h and perforation rate (OR 0.79; 95% CI 0.51–1.22; *p* = 0.28) or complication rate (OR 0.68; 95% CI 0.39–1.17; *p* = 0.16).

## Discussion

This retrospective study of 1,027 patients with suspected complicated appendicitis was conducted to identify risk factors for complications and to determine a safe window for surgical intervention. To our knowledge, this represents the largest single-center cohorts to only include patients with suspected complicated appendicitis reported to date.

Appendicitis affects individuals across all age groups. In our cohort, the mean patient age was 49.3 years, with 24.4% of patients aged over 65. Most patients with complicated appendicitis presented after experiencing symptoms for more than 24 h. Histopathological analysis confirmed perforation in 47.5% of all cases indicating the precision of preoperative diagnostics.

The majority of patients underwent laparoscopic appendectomy. The conversion rate to open surgery was 14.4%, which is significantly higher than that observed in patients with uncomplicated appendicitis, highlighting the increased surgical complexity in these cases [16, 17].

Multivariable logistic regression identified age over 65 years, elevated body mass index (BMI), symptom duration exceeding 48 h, and elevated inflammatory markers (white blood cell count > 15/nl and C-reactive protein > 150 mg/l) as independent risk factors for perforation. Advanced age and high BMI may reflect the presence of comorbid conditions that contribute to delayed diagnosis and treatment. Diagnostic methods, such as ultrasound imaging are challenging in obese patients and may lead to a delay in diagnosis. Furthermore, model studies suggest that obesity leads to delayed appraisal as obese patients may avoid seeking healthcare due to stigma [18].

The mean time from hospital admission to surgery was 6.8 h, with 87.9% of patients undergoing surgery within 12 h. Notably, logistic regression analysis revealed that surgical delays exceeding 6 h were not associated with an increased risk of perforation or complications, even in cases of suspected complicated appendicitis. The literature provides evidence that an in-hospital delay of 24 h is safe in cases of uncomplicated appendicitis [6, 11]. Our results suggest that, in patients with advanced appendicitis, the urgency of surgical intervention may be less critical than previously believed. Analyzing only suspected complicated cases resolves the bias of earlier treatment in more critically ill patients with possible perforation. Because clinical findings, such as abdominal guarding, high inflammatory values, and radiologic findings suggestive of perforation, influence the decision to operate early on a patient, we performed a propensity score-matched analysis in addition to the logistic regression analysis to account for differences between patients with a shorter in-hospital delay and those with a longer delay, thereby further reducing selection bias. Propensity score matching confirmed that a waiting more than six hours for surgery is safe with regards to complication rate and perforation. Complication rates did not significantly differ between both groups.

Propensity score matching was repeated for an even longer waiting time for surgery of more than 9 h. Similarly to what was already observed, complication and perforation rate did not increase with increased time to surgery.

Therefore, our findings suggest that a surgical delay of approximately 6 to 9 h does not appear to adversely affect short-term outcomes. Nonetheless, it is important to acknowledge that long-term complications were not assessed in this study. In particular, the incidence of small bowel obstruction and incisional hernias warrants further investigation, as these have been reported in a substantial proportion of patients with complicated appendicitis [19].

The data indicate that the timing and duration of symptoms play a critical role in the development of complicated appendicitis. However, the literature also suggests that complicated and uncomplicated appendicitis may represent distinct pathological entities rather than progressive stages of the same disease thus challenging the concept linear progression from uncomplicated to complicated appendicitis with time [20–22].

The results of this study suggest that the risk of perforation and perioperative complications is influenced by a variety of factors, with age, BMI, symptom duration, and preoperative inflammatory markers playing a key role. Interestingly, waiting time before surgery did not significantly affect the outcomes, which may challenge the commonly held belief that immediate surgery is critical to avoid complications. Our data suggest that a delay in appendectomy of 6 to 9 h can be considered acceptable. This finding may support triage decisions during periods of high surgical emergency workload and assist in determining whether patient transfer is necessary due to limited capacity or expertise.

A retrospective study design was chosen due to ethical concerns associated with deliberately delaying surgical intervention in cases of complicated appendicitis. However, the data presented here provide a valuable foundation for the development of a prospective study in which patients are not excluded on the basis on radiologic, clinical and laboratory findings, as was the case in the prospective PERFECT and DELAY trials, allowing a more robust investigation of delayed surgery in acute appendicitis [6, 10].

Nevertheless, the treatment of patients with complicated appendicitis remains challenging, with individual risk factors and clinical judgment playing a crucial role. It should be borne in mind that the clinical condition of patients with appendiceal perforation can deteriorate rapidly, in the presence of other comorbidities. Therefore, when considering a delay in surgical treatment in patients with suspected perforation or complicated appendicitis, a timely and repeated clinical reassessment of the patient’s condition and antibiotic treatment is required.

In addition, it should be kept in mind that advanced disease usually causes severe discomfort and that unnecessary delay in surgery should be avoided at all costs to minimize pain for the patient.

This study has several important limitations that must be acknowledged. First, as with any retrospective analysis spanning an extended study period, incomplete documentation of individual variables represents an inherent limitation that could not be entirely avoided. Second, formal intraoperative grading of appendicitis severity (e.g., using WSES or AAST classification systems) was not systematically documented across the entire study period (2008–2023). Consequently, the reference standard for the primary outcome “perforation” was histopathology alone, rather than a combined surgical and histopathological assessment. While histopathological evaluation is widely accepted as the most specific method for severity classification, future prospective studies should incorporate standardized intraoperative documentation to allow a combined reference standard.

Conversely, several strengths of this study deserve emphasis. The near absence of exclusion criteria and the use of real-world data from a high-volume center enhance the external validity of our findings. Furthermore, as ultrasound served as the primary diagnostic modality — consistent with clinical practice across a broad range of healthcare systems worldwide — our results are likely generalizable beyond settings that rely predominantly on CT-based primary imaging, which represents the majority of published literature in this field.

## Conclusion

This large retrospective cohort of patients with suspected complicated appendicitis identified advanced age, BMI, prolonged symptom duration and elevated inflammatory markers as independent risk factors for perforation and complications. Propensity score matched analysis revealed that in-hospital delays of up to 6–9 h are not associated with increased perforation and/or complication rates thus challenging the notion that immediate surgery is imperative in all cases of suspected complicated appendicitis. Nonetheless, careful patient selection, close clinical monitoring, and timely reassessment remain essential.

## Supplementary Information

Below is the link to the electronic supplementary material.

Supplementary Material 1 (DOCX 25.1 KB)

Supplementary Material 2 (DOCX 25.3 KB)

## Data Availability

Data is provided within the manuscript or supplementary information files. A fully anonymized data set and the statistical code can be made available upon justified scientific request and after ethical approval has been granted. Depending on the extent of the data use and the planned research, either appropriate credit or co-authorship must be granted to the authors of this study. Any requests should be addressed to the corresponding author.
