# Supplementary material for: Safety of in-hospital delay of appendectomy in complicated appendicitis – a propensity score–matched analysis of 1027 patients
Source: Langenbecks Arch Surg. 2026 Oct 23;411(1):288. doi: 10.1007/s00423-026-04264-3 (PMC13694433; doi:10.1007/s00423-026-04264-3)
Supplement: Supplementary file 1 [file 423_2026_4264_MOESM1_ESM.docx]

|  | Time to surgery ≤ 9h | Time to surgery > 9h | p | SMD |
| --- | --- | --- | --- | --- |
|  | n=773 | n=179 |  |  |
| Age | 48.7 (19.6) | 49.0 (19.2) | 0.876 | 0.013 |
| Leukocyte count > 15/nl | 340 (44.0%) | 60 (33.5%) | 0.013 | 0.216 |
| CRP level > 150mg/l | 354 (45.8%) | 87 (48.6%) | 0.551 | 0.056 |
| Duration of symptoms |  |  | 0.122 | 0.226 |
| 0-12h | 48 (6.2%) | 16 (8.9%) |  |  |
| 12-24h | 126 (16.3%) | 31 (17.3%) |  |  |
| 24-48h | 193 (25.0%) | 33 (18.4%) |  |  |
| 48-72h | 146 (18.9%) | 27 (15.1%) |  |  |
| more than 72h | 260 (33.6%) | 72 (40.2%) |  |  |
| Sex  male  female | 414 (53.6%)  359 (46.4%) | 75 (41.9%)  104 (58.1%) | 0.006 | 0.235 |
| Abdominal guarding | 444 (57.4%) | 63 (35.2%) | <0.001 | 0.458 |
| ASA score | 1.75 (0.74) | 1.82 (0.75) | 0.261 | 0.093 |
| BMI | 26.8 (5.4) | 26.8 (6.7) | 0.997 | <0.001 |
| Suspected perforation on imaging | 326 (42.2%) | 67 (37.4%) | 0.281 | 0.097 |
| Diabetes | 32 (4.1%) | 12 (6.7%) | 0.202 | 0.113 |
| Previous abdominal surgery | 113 (14.6%) | 34 (19.0%) | 0.179 | 0.117 |
| Date are mean ± SD or n (%)  SMD standard mean difference, BMI body mass index, ASA American Society of Anaesthesiologists, CRP C-reactive protein | | | | |

Table 1a Baseline characteristics before matching in patients with CRP≤100mg/l and / or suspected perforation for patients with an in-hospital delay ≤9 hours and >9 hours
