# Supplementary material for: Safety of in-hospital delay of appendectomy in complicated appendicitis – a propensity score–matched analysis of 1027 patients
Source: Langenbecks Arch Surg. 2026 Oct 23;411(1):288. doi: 10.1007/s00423-026-04264-3 (PMC13694433; doi:10.1007/s00423-026-04264-3)
Supplement: Supplementary file 2 [file 423_2026_4264_MOESM2_ESM.docx]

|  | Time to surgery ≤ 9h | Time to surgery > 9h | p | SMD |
| --- | --- | --- | --- | --- |
|  | n=178 | n=178 |  |  |
| Age | 49.0 (19.8) | 49.0 (19.3) | 0.991 | 0.001 |
| Leukocyte count > 15/nl | 58 (32.6%) | 60 (33.7%) | 0.910 | 0.024 |
| CRP level > 150mg/l | 81 (45.5%) | 87 (48.9%) | 0.596 | 0.068 |
| Duration of symptoms |  |  | 0.943 | 0.093 |
| 0-12h | 19 (10.7%) | 16 (9.0%) |  |  |
| 12-24h | 26 (14.6%) | 31 (17.4%) |  |  |
| 24-48h | 33 (18.5%) | 33 (18.5%) |  |  |
| 48-72h | 29 (16.3%) | 27 (15.2%) |  |  |
| more than 72h | 71 (39.9%) | 71 (39.9%) |  |  |
| Sex  male  female | 73 (41.0%)  105 (59.0%) | 75 (42.1%)  103 (57.9%) | 0.914 | 0.023 |
| Abdominal guarding | 61 (34.3%) | 63 (35.4%) | 0.911 | 0.024 |
| ASA score | 1.79 (0.75) | 1.81 (0.75) | 0.778 | 0.030 |
| BMI | 26.9 (5.9) | 26.8 (6.7) | 0.983 | 0.002 |
| Suspected perforation on imaging | 68 (38.2%) | 67 (37.6%) | 1.0 | 0.012 |
| Diabetes | 7 (3.9%) | 11 (6.2%) | 0.468 | 0.103 |
| Previous abdominal surgery | 34 (19.1%) | 33 (18.5%) | 1.0 | 0.014 |
| Date are mean ± SD or n (%)  SMD standard mean difference, BMI body mass index, ASA American Society of Anaesthesiologists, CRP C-reactive protein | | | | |

Table 1b Baseline characteristics after matching in patients with CRP≤100mg/l and / or suspected perforation for patients with an in-hospital delay ≤9 hours and >9 hours
